# Registered nurses’ perceptions and experiences of autonomy: a descriptive phenomenological study

**DOI:** 10.1186/s12912-019-0378-3

**Published:** 2019-11-01

**Authors:** Titilayo Olufunke Oshodi, Benjamin Bruneau, Rachel Crockett, Francia Kinchington, Shoba Nayar, Elizabeth West

**Affiliations:** 1 Anglia Ruskin University, Faculty of Health, Education, Medicine, and Social Care, Chelmsford Campus, William Harvey Building, Bishop Hall Lane, Chelmsford, Essex CM1 1SQ UK; 2 University of Greenwich, Faculty of Education and Health, Southwood Site, 2nd Floor Seacole Building, Avery Hill Road, London, SE9 2UG UK; 3 Division of Psychology, Faculty of Natural Sciences, University of Stirling, Stirling, Scotland FK9 4LA UK; 4 University Teaching Fellow, University of Greenwich, Faculty of Education and Health, Mansion Site, London, SE9 2PQ UK; 5 Applied Social Science, University of Greenwich, Faculty of Education and Health, Southwood Site, 2nd Floor Bronte Building, Avery Hill Road, London, SE9 2UG UK

**Keywords:** Hospital, Nurse, Autonomy, Autonomous practice, Descriptive phenomenology, Qualitative

## Abstract

**Background:**

Professional autonomy is a key concept in understanding nurses’ roles in delivering patient care. Recent research exploring the role of autonomy in the nursing work environment indicated that English and American nurses had differing perceptions of autonomy. This qualitative study aimed to explore the understanding and experiences of autonomy of nurses working in England.

**Methods:**

A descriptive phenomenological analysis of data from 48 semi-structured interviews with registered nurses from two National Health Service (NHS) hospitals (purposive sample) was used to explore the concept of autonomy.

**Results:**

Six themes were identified: *working independently; working in a team; having professional skills and knowledge; involvement in autonomy*; *boundaries around autonomy;* and *developing autonomy requires support*. A key finding was that nurses related autonomy to their clinical work and to the immediate work environment of their ward, rather than to a wider professional context. Nurses also perceived that autonomy could be turned off and on rather than comprising an integrated aspect of nursing.

**Conclusions:**

Findings suggest that nurses in England, as framed by the sample, had a local ward-focused view of autonomy in comparison to nurses in America, who were reported to relate autonomy to a wider involvement in hospital level committees. Findings further indicate that autonomy was practiced occasionally, rather than incorporated into practice. Findings highlight the need for nurses in England to adopt a broader perspective and actively contribute to writing hospital guidelines and policies that recognise the importance of autonomy to nurse training and practice.

## Background

The concept of autonomy has been an important topic of study in the nursing profession for many decades and has given rise to a range of definitions. Skar [1] defined professional autonomy as “having the authority to make decisions and the freedom to act in accordance with one’s professional knowledge base” (p. 2226). Kramer and colleagues [2] delineated three dimensions of autonomy in clinical practice settings. The first is *clinical or practice autonomy* which refers to independent, interdependent, and accountable decision making by nurses for the primary and immediate benefit of the patient. The second dimension is *control over nursing practice autonomy*, or organisational autonomy, which relates to the regulation and the development of policies for nursing by nurses. The third is *job or work autonomy*, which describes unit-level-group decision making for the purpose of organising the work day and setting priorities among tasks. Gagnon and colleagues [3] stated that individual, clinical, organisational, and professional autonomies have been identified in the literature and, in some cases, have been used interchangeably. They cautioned that these terms are not synonymous, even though they share similar features such as responsibility and accountable decision-making. Kramer and colleagues [2] further warned that the impact of autonomy on patient outcomes cannot be determined when various concepts of autonomy are labelled the same but differ in meaning and are measured with tools or instruments that do not fit the concept. The need for clear, shared understanding of the meaning and an understanding of the empirical measurement of autonomy is critical.

An American study [4] of 570 acute care hospitals found that patients receiving care within hospitals that promote nurse autonomy have lower risk for, and complications leading to, death within 30 days. Van Oostveen and Vermeulen [5] stressed that the study carried out by Rao and colleagues [4] provides evidence that when nurses do not have the ability to exercise their clinical and organisational knowledge, patient safety is put at risk. Health organisations are responsible for providing necessary means for nurses to act autonomously by formulating clear roles, responsibilities and behaviours, and enhancing competence in practice and decision-making [5].

Previous research [6], explored the relationship between the nursing work environment and nurse assessed quality of care using the Essentials of Magnetism II (EOMII) Scale [7], a measure developed in America which conceptualises autonomous nursing practice as a key element of a good quality work environment for nurses. This research suggested that the way in which nurses in England conceptualise autonomy may differ from that of nurses working in America. Bai, Hsu, and Zhang [8] explored the psychometric proprieties of the EOMII scale. One of the extracted factors, *Restriction of Decision-making*, suggested that Chinese nurses experience prohibitions on autonomous decision-making. The authors recommended further clarifications of the definitions and scope of autonomy in Chinese clinical settings. De Brouwer and colleagues [9] also assessed the psychometric properties of the EOMII amongst nurses in The Netherlands. Their findings suggested that the Dutch respondents used different definitions of autonomy which could have affected the way they answered items in the clinical autonomy subscale. One item in particular, stated that nurses have to have permission before practising autonomously. This includes the premise that a nurse is only able to practise autonomously after asking for permission. The authors suggested that how respondents interpret the item determines whether they perceive the item positively or negatively. De Brouwer and colleagues [9] recommended further research defining clinical autonomy by non-US nurses.

Labrague, McEnroe-Petitte, and Tsaras [10] found that consistent with international studies, nurses in the Philippines demonstrated moderate levels of professional autonomy. Nurses who had higher levels of autonomy tended to be high performing, satisfied, and committed in their jobs. Organisational efforts are critically important to foster autonomy in practicing nurses through adequate support, education, training, and developed policies [10].

In a qualitative study carried out in Iran, [11] identified two main barriers to gaining professional autonomy, namely, profession-related and organisational. Profession-related barriers included lack of strong professional bodies and capacity to exercise autonomy. Organisational barriers included role ambiguity, a directive rather than supportive workplace, and lack of motivation. AllahBakhshian and colleagues [11] highlighted historical gender issues and greater number of women in nursing, autocratic leadership styles, and doctor-led management models in Iran as reducing nurses’ self-esteem and discouraging nursing autonomy.

With an increasing internationally mobile work force, discrepancy between conceptualisations of autonomy held locally and by non-local nurses have the potential to impact the delivery of patient care.

## Methods

A phenomenological approach was employed to understand the perceptions and experiences of autonomy of nurses in England. Phenomenology seeks to understand people’s everyday life experiences [12] by revealing what lies ‘hidden’ in them [13]. Phenomenology is interested in the activities of consciousness and the objects that present themselves to the conscious [14]. Data were collected through semi-structured interviews [15], and the subsequent analysis utilised Giorgi, Giorgi, and Morley’s [16] method of descriptive phenomenological data analysis to explore the concept of autonomy.

Given the literature that indicates differences in how autonomy is conceptualised among nurses internationally, descriptive phenomenology was chosen for its emphasis on the ‘pure’ description of people’s experiences [13], and not based on the researchers’ interpretation of people’s descriptions of their experiences. Giorgi [14] argued that a larger part of phenomenology is descriptive, but this does not rule out phases where interpretations also take place. Phenomenology does not dictate the phenomena; rather seeks to understand how phenomena present themselves to consciousness and the elucidation of this process is a descriptive task. Giorgi [14] highlighted the difference between description and interpretation in that description is an acknowledgement that there is a “given” that needs to be described precisely as it appears and nothing to be added to it nor subtracted from it. Interpretation is the adoption of a non-given factor to help account for what is given in experience such as a theoretical stance, a hypothesis, or an assumption. Giorgi [14] specified that a researcher who wants to employ the descriptive phenomenological psychological method has to firstly assume the attitude of the phenomenological reduction, whereby the researcher must resist from positing or hypothesising as existing, whatever object or state of affairs is present to her. It is also important that the researcher refrains from bringing in non-given past knowledge to help account for whatever she is presented with [14]. The researcher concentrates on the “given” as a phenomenon, and everything that is said about the phenomenon is based upon what is given [14].

### Aims

The overarching goal of this qualitative study was to explore how nurses in England conceptualise autonomy and how they put the concept into practice. Specifically, the study asked:

- RQ1: How do registered nurses in England understand the concept of autonomy in practice?
- RQ2: What are the experiences of nurses in England of autonomy in practice?

### Ethical considerations

The study received ethics approval from London-Surrey Borders National Health Service (NHS) Research Ethics Committee, study reference number 11/LO/1329. Participants’ anonymity and confidentiality were protected.

### Setting

The study was conducted in two National Health Service (NHS) Trusts in the South East of England. A total of 28 wards were included in the study; 11 wards in hospital A, and 17 wards in hospital B. Hospital A is a District General Hospital which serves a population of around 300,000 and has around 500 in-patient beds. Hospital B is a general hospital which serves a population of about 400,000 and has approximately 600 beds.

### Participants

Registered nurses providing direct adult patient care on 29 wards were invited to participate in the study; however, one ward manager in Hospital A declined participation on behalf of her nurses. Nurses eligible to participate were those who had worked on their present wards for a minimum of one month. Twenty-six registered nurses were interviewed from hospital A and 22 from hospital B. Participants comprised 13 ward managers, 11 ward sisters, two charge nurses and 22 staff nurses with different levels of nursing experience and different grade levels in nursing. Forty-five respondents were female, more than a quarter were aged between 35 and 39 years, and 23 worked on the surgical specialities, while the remaining worked on medical specialities.

### Recruitment process

Maximum variation sampling was used in recruiting the participants for the purpose of achieving comparability [17], as participants varied in professional and socio-demographic characteristics. Maximum variation sampling is a purposive sampling procedure based on achieving representativeness or comparability [18]. Participants were chosen because they had particular characteristics such as experience and roles which would enable understanding of the central themes under investigation. The participants included staff nurses, sisters, charge nurses and ward managers. They had different levels of nursing experiences and were of different grade levels in nursing. Selecting samples with diverse characteristics would highlight the similarities or diversity in their views.

Between June and July 2013, the first author contacted the ward managers of the participating wards by telephone and booked appointments to discuss the plan to conduct qualitative interviews with the registered nurses. In July 2013, the author visited the 28 wards and discussed with the ward managers the aims, plans and the purpose of the interviews and when to contact the nurses. The ward managers agreed to inform the nurses about the interviews during shift handovers and ward meetings.

Sixty-five registered nurses were approached for the interview, seven declined to be interviewed. The 58 nurses who agreed to participate were later contacted and suitable times for the interviews were arranged. At this point, five nurses were unable to participate because they were too busy with patient care, and five were not interviewed because data saturation was reached during the 48th interview.

### Data collection

Forty-eight registered nurses were interviewed by the first author in July 2013 using semi-structured interviews which lasted between 6 and 18 min to enable nurses on duty, or on their breaks during shifts, to participate without having to give up substantial periods of their limited time. The interviews took place in quiet rooms on the wards. Participants gave their informed consent and agreed for the interviews to be digitally audio-recorded. Each participant was informed that the data might appear in published work and assured of anonymity. An interview schedule containing four questions was developed in response to the aims of the study and was used to guide the interviews (Table 1). All interviews started with a standard introduction about the study and then moved to the broad aim of the research [19]. The questions were planned but flexible [19] so that when required, the researcher altered the sequence of questions and probed for more information [20] to tease out strands of participants’ narratives to complete the story [21].

**Table 1:** Interview schedule

Interview schedule	
(1) Can you tell me what you understand by autonomous nursing practice please?	
(2) When nurses say “autonomous nursing practice”, what does that mean to you?	
(3) Can you give an example of an incident in your practice that was autonomous?	
(4) Have you any comment or is there anything that you think is missing that is autonomous practice?	

### Data analysis

All interviews were transcribed verbatim and analysed using the five-step descriptive phenomenological analytical method described by Giorgi, Giorgi, and Morley [16]. In the first step, the transcripts were read repeatedly to gain a sense of the whole situated description. Prior to reading the data, the researchers met to discuss their understandings of autonomy and the importance of putting aside any preconceptions, a situation described as bracketing [22].

In the second step, transcripts were read with an attitude of scientific phenomenological reduction. That is, the objects that emerged within the description were taken to be the phenomena comprising the whole experience.

The third step entailed the researcher breaking the narrative into parts. As transcripts were read, slashes were placed in the description to mark a new meaning as lived by the participants. A table was created (Table 2) where the first column represents the meaning units using the language of the participants.

**Table 2:** Development of themes and sub-themes (an illustration)

Meaning unit	Condensed meaning units	Subthemes	Themes
*“I feel that they [nurses] probably don’t have that full understanding of what it means. They know that they are needing to work within their Code of Conduct… I think they’re also aware of the decisions that they need to make and they are aware of the word, but I think that they find it very difficult to describe it in use in practice...I think it’s something that they probably automatically do but don’t really think ‘Ah this is what I am doing’ and put a name to actually being autonomous in their practice”* (P38 WM:16y)	Autonomy is implied	4.1 Autonomy is experienced on a daily basis	4.Involvement in autonomy
“*...if there’s no need for them to have IV (intravenous) fluids running and they’re eating and drinking and then you can make a decision to stop the IV fluids*” (P22 SN:5y).	Autonomy experienced on a daily basis		
“*I think autonomous is just like you do it routine.... it’s like you come to work, you wash the patient… you give them medication, you take your observation and make things comfortable*” (P24 SR:22y).	Autonomy is routine work		
“*...my junior sister would make a decision to take out a central line, to take out a catheter and to move a patient onto diet and fluids without referring to a doctor over a weekend*” (P18 WM:18y).	Situational autonomy	4.2 Demonstrating autonomy in exceptional circumstances	
“*I would probably take more of an autonomous role of a weekend in a way, of that leadership… the sisters of a week kind of run the shift don’t they, or the nurse in charge*” (P21 SN:2y9m).	Situational autonomy		

In step four, participants’ expressions were transformed into psychological meanings lived by the participants which, in some instances, necessitated that the original expressions of the participants be changed. Transformations were also intended to generalize the meanings to enable integration with other descriptions. The second column in Table 2 represents the transformed expressions, i.e. condensed meaning units.

Finally, in step five, the transformed meaning unit expressions were used as the basis for describing the general psychological structure of the experience, i.e. themes. This was done by reviewing all of the transformations written in the second column to determine the essential structure or themes. These were then used to clarify and interpret the raw data. Following this final refinement, six themes and seven subthemes were identified, as presented in Table 3.

**Table 3:** Themes and subthemes

Themes	Subthemes
1. Working independently	
2. Working in a team	
3. Having professional skills and knowledge	• Having the right skills and knowledge • Decision-making based on clinical judgement • Making informed and evidence-based decisions
4. Involvement in autonomy	• Autonomy is experienced on a daily basis • Demonstrating autonomy in exceptional circumstances
5. Boundaries around autonomy	• Working within boundaries • Working beyond boundaries
6. Developing autonomy requires support	

### Rigour

The first author conducted all 48 interviews, posing the questions the same way to all the participants. Five authors who were specialised in qualitative research independently went through the transcripts, confirmed the accuracy of the meaning units, as well as the transformed expressions. All the researchers agreed on the final themes.

## Results

Six key themes emerged from the data analysis: *working independently; working in a team; having professional skills and knowledge; involvement in autonomy*; *boundaries around autonomy;* and *developing autonomy requires support*. Each theme and accompanying sub-themes are examined in turn and illustrated by quotations. Codes are used after each participant quote to represent information relating to their designations and years of experience. Examples of the codes and their interpretations are presented in Table 4.

**Table 4:** Interviewee codes

Code	Interpretation
P1(SN:14y)	Participant 1, Staff Nurse, 14 years of nursing experience
P10(SN:8 m)	Participant 10, Staff Nurse, eight months of nursing experience
P3(SR:33y)	Participant 3, Ward Sister, 33 years of nursing experience
P27(CN:15y)	Participant 27, Charge Nurse, 15 years of nursing experience
P39(WM:25y)	Participant 39, Ward Manager, 25 years of nursing experience

## Theme 1: working independently

Participants defined their understanding of autonomy as: nurses’ ability to work on their own without external influence. Participants stated that working on their own, required a readiness to act on their own initiative. For instance, typical descriptions of autonomy included comments such as: “*being able to work independently*” (P6 SN:3½y) and “*autonomy is working on your own*” (P5 WM:43y). Some participants linked autonomous nursing practice to working without supervision or using self-directed guidance. The ability to work free from other practitioners’ control or direction was emphasised and exemplified by the following comments: “… *they’re not given their direction from somebody, so they can work independently, on their own*” (P47 SN:10y) and “*To me it is the ability to be able to work under your own guidance… and prepare your day outside of a team*” (P28 WM:17y)*.*

However, to work independently requires a degree of self-assurance. Participants identified confidence as being connected with autonomous practice. For instance, P15(SR:5y) commented: “*...they [autonomous practitioners] are responsible and having the confidence to do things on your own without having to constantly seek help and advice from others or relying on others to do it for you*”. In addition to participants’ beliefs that working on their own required confidence, they related accountability and responsibility to their ability to work independently. The participants mentioned the corollary to working independently which is that one is then accountable for the actions taken and responsible for the results. It was noted that issues of accountability and responsibility arose mostly during the interviews with the ward managers and the ward sisters. For example, one ward manager reflected that:

*...autonomous practice is working independently and being accountable for your own actions... They’re doing it on their own back if they’re not being told what they need to do first.* (P40 WM:7y)

Participants also linked working on their own with risk and acceptance of uncertainty, acknowledging the risks that are associated with autonomous action. The participants perceived risk as the likelihood of an event happening with potential beneficial or harmful outcomes for the patients or themselves, with respect to their jobs. This can be gathered from the responses of participants such as P34(WM:11y) who perceived autonomous action to be linked to the likelihood of risk: “*...obviously comes with an element of risk when dealing with patients, but it’s being able to evaluate and weigh all that risk and make all the right choices for your patients...*”; and P1(SN:14y) “*...that’s why sometimes being independent, having independent autonomous, it can create trouble and then you will feel that fear… but at the same time you have to take risk.*”

Participants perceived autonomy as being both restricted to working on their own and encompassing working within a team.

## Theme 2: working in a team

Participants were clear that nurses also work as part of a team. Many of the participants perceived autonomy as working and making decisions within the context of a team, with typical comments such as: “*...you’re working, obviously as part of a team*” (P10 SN:8 m); and “*…making my own decisions, obviously within the context of everyone I am working with, as being part of the team*” (P9 SN:23y). These comments highlighted participants’ emphasis on the importance of collaboration as enabling team members to work more closely together to make decisions. The emphasis placed on team involvement as a key ingredient in autonomy, can be gathered from comments such as: “*...but it has to be into a team as well... at times we have to wait for their decision too*” (P3 SR:33y); and “*…you always involve the team*” (P1 SN:14y).

Participants identified that within the team members may have a range of complementary skills to support and assist one another and improve individual performances. They also described teamwork as a support system through contributions of their practice and knowledge to the multidisciplinary team, or through reliance on the team as guidance, as can be gathered from the following comments: “*…how you will demonstrate your practice and how you contribute your knowledge to the other team, MDT [Multi-Disciplinary Team]…*” (P41 SN:20y); and “*…it’s good to have guidance as well from team members and yeah... I must admit sometimes I am not very assertive, and I do rely on my colleagues*” (P31 SN:8y). One participant reflected on the inter-relational nature of nursing work. This ward sister stated that autonomy for nurses is “*…to be working in one scope of proficiency and knowledge and skill framework, to be able to practice independently but also be able to seek support from seniors, doctors”* (P16 SR:4y).

Autonomy in the context of teamwork revealed how nurses work interdependently, utilising and/or sharing their knowledge and skills. In addition to teamwork, participants emphasised the skills and knowledge required for autonomy.

## Theme 3: having professional skills and knowledge

This theme depicts the professional skills and knowledge that nurses require for autonomy, and is comprised of three subthemes, namely having the right skills and knowledge; decision-making based on clinical judgement; and informed and evidence-based decisions.

### Subtheme: having the right skills and knowledge

Nurses were aware of the importance of their skills, knowledge, experience, and competence in relation to working independently, making complex decisions and managing patient care. This gave them the ‘support and backing’ to be autonomous practitioners. Participants described the use of nursing knowledge and skills as important ingredients in autonomy: “*…in my eyes it is your basic nursing skills and your knowledge*” (P14 SR:41y).

Participants further highlighted the ability to be able to work independently with the use of existing skills and knowledge to make decisions, as described by P16(SR:4y): “*…relying on my own skills and knowledge, I am able to make those basic decisions and some more complex decisions.*”

One of the participants gave an example of how decisions are made in practice based on nursing knowledge. This participant emphasised that nursing knowledge and skills are required in order to make decisions such as choosing the right dressings, and doing drug administration:

*Their drug rounds are autonomous. They very rarely have to come to me. They have their BNF [British National Formulary] if they need back-up. Dressings they do, that’s autonomous, but obviously they must have the knowledge to be able to choose the right dressings.* (P39 WM:25y)

Some participants also described autonomous nursing practice as being linked to nurses having the confidence to use their knowledge and experience to make decisions. Comments such as: “*Staff having the confidence and experience and knowledge…*” by P7(WM:33y); and “*…it’s nurses using their own knowledge and experience to manage patient care*” (P43 WM:10y) exemplify this finding.

Some participants described autonomous nursing practice as being linked to decision making based on level of experience. This was because autonomy was viewed as a process that developed over time, through the experience of nursing practice: “*...we work autonomously when we are interpreting observations... but if they are abnormal then some nurses with more experience might do something differently*” (P43 WM:10y).

In a similar vein, another participant perceived autonomous practice as synonymous with a certain level of experience, stating:

*...once you have got a certain level of experience you can work autonomously within your group of patients… and more junior nurses, you would obviously expect them to use less autonomy than somebody with more experience.* (P47 SN:10y)

This was corroborated by a ward manager:

*...she might have autonomy in one area of her practice but she may not have it in another, she may defer to somebody else, so it is dependent on your experience.* (P18 WM:18y)

When participants were asked to provide examples of autonomy in practice, a ward manager commented that this meant that nurses make decisions on their own in specific situations, and provided the following example in practice:

*it’s when say one of my nurses are working on a patient with non-invasive ventilation and they make the decision to adjust the patient’s settings based on their response to the treatment... based on their own education and their own practices and experiences.* (P35 WM:15y)

Another participant linked nursing knowledge and skills with competence, highlighting that nurses feel supported in being autonomous when they are equipped with the right competence and skills:

*by ensuring that nurses are equipped with the right competence and the right skills gives them that support and the backing for them to be autonomous…* (P19 WM:8y)

Participants recognised the need for training: “…*the more training you have like to back you up, it’s very good*” (P31 SN:8y). One participant commented on the availability and usefulness of training as being: “*I do think autonomy is really important and I don’t think we do get enough of it in our training. I do think that we need …have an understanding in terms of patients’ treatment*” (P42 SN:6 m).

Understanding that additional training regarding autonomy would support nurses, and in turn result in better patient care, other participants highlighted inadequate training as a factor hindering autonomous nursing practice. This view was expressed by a participant who believed that: “*...provided that we get …the level of training and the level of exposure we can deliver good patient care*” (P48 SR:17y). Another participant pointed out how difficult it is to have access to courses: “*...sometimes it can be hard to get on all the courses that you perhaps want to because of ward pressures, but without having that knowledge, sometimes these decisions are perhaps not safe decisions, if your knowledge isn’t up-to-date*” (P29 SN:9y).

A ward manager argued that due to the oversimplification of aspects of the nursing education or role, the intellectual standards of the nursing profession or education are undermined. She commented:

*...I think that we have dumbed down nursing/nurse training, I think a lot of the stuff that we see as extended practice, things like cannulation, phlebotomy, OK it’s task-orientated but it is actually improving your patient’s care, we have made a big thing of, and it's become an add-on. It's not, it's basic nursing care, it's what we do, and I actually feel that we have dumbed down nursing to some extent...* (P18 WM:18y)

This perception appeared to be shared by another ward sister who commented that autonomous practice is an important element that should be focused on during nursing training:

*... I think autonomous practice is very important for nurses. I think it needs to be something that’s focused on in the nurse training. I think the trainee nurses are very well supported but I think sometimes we don't allow them to think for themselves and to act for themselves. So I think that’s an important element to maybe take back to the basics in nurse training...* (P16 SR:4y)

### Subtheme: decision-making based on clinical judgement

Participants described clinical judgement as one of the skills nurses draw upon while making clinical decisions. They viewed clinical judgement as a key attribute of professional practice, central to safe and effective care, as it enables nurses to distinguish between bad and good decisions based on knowledge: “*...you’re taking your own clinical judgment and knowledge to make the decisions that you’re making*” (P10 SN:8 m). This statement was corroborated by other participants who perceived autonomy as: “*making my own clinical judgements*” (P44 SR:18y), or when “*I am making decisions based on my clinical judgement and my experience*” (P35 WM:15y).

Another participant talked of how clinical judgement is being utilised in practice to escalate patients without going to superiors:

*…if they come across a patient who is unwell, they know how to escalate that without having to go to someone more senior. Yeah, they make judgements…* (P7 WM:33y)

Clinical judgement was also linked to the ability to make logical rational decision based on the observation of the patients: “*…whether or not you need to put IV [intravenous] fluids up, so then they’re prescribed but the patient may not necessarily need it, you are using your clinical judgement*” (P10 SN:8 m).

In addition to describing clinical judgment as an attribute required for autonomy, participants highlighted the ability to make informed and evidence-based decisions as important in autonomous nursing practice.

### Subtheme: making informed and evidence-based decisions

Participants described autonomy as the ability to make evidence-based decisions such as being dependent on the availability of the best, up-to-date knowledge and research, without which practice would be unsafe: “*…but without having that knowledge, sometimes these decisions are perhaps not safe decisions, if your knowledge isn’t up-to-date*” P29(SN:9y)*.* Furthermore, two ward managers emphasised the importance of utilising information in making safe and informed decisions, and reiterated the corollaries of autonomy which are accountability and responsibility: “*to make informed decisions and take responsibility for them...*” (P7 WM:33y), and “*you are accountable for your responsibility of the information that you use for practice*” (P38 WM:16y).

Some participants were more specific, referring to both informed decisions and, importantly, to decisions based on scientific knowledge. This finding was evidenced in comments such as: “*...making my own clinical judgements and decisions on an evidence base*” (P44 SR:18y); and “*...so autonomy to me means being able to make your own evidence-based decisions in practice based on the best knowledge and research that’s available to you*” (P34 WM:11y).

In addition to the discussion of the skills and knowledge required for autonomy, participants considered the importance of decision making either on a day-to-day basis or in emergency situations.

## Theme 4: involvement in autonomy

This theme is specifically about participants’ experiences of autonomy. Two subthemes were identified: autonomy is experienced on a daily basis and demonstrating autonomy in exceptional circumstances.

### Subtheme: autonomy is experienced on a daily basis

Day-to-day autonomy, as demonstrated by nurses on every shift, was identified as a component of the nursing job based on nursing knowledge and linked to everyday routine and procedures. As a result of autonomy being expressed through everyday tasks, participants revealed how autonomy is implied rather than overtly expressed. A ward manager stated that nurses would be unable to explicitly define autonomy in reference to their own practice. She maintained that autonomous practice was something that nurses do automatically without actually thinking about it:

*I feel that they [nurses] probably don't have that full understanding of what it means. They know that they are needing to work within their Code of Conduct… I think they’re also aware of the decisions that they need to make and they are aware of the word, but I think that they find it very difficult to describe it in use in practice...I think it’s something that they probably automatically do but don't really think ‘Ah this is what I am doing’ and put a name to actually being autonomous in their practice.* (P38 WM:16y)

Some participants equated autonomous practice to routine tasks such as washing patients and dressing wounds: “*I think autonomous is just like you do it routine.... it’s like you come to work, you wash the patient… you give them medication, you take your observation and make things comfortable*” (P24 SR:22y).

Besides describing autonomous practice as routine tasks, one participant linked it to procedural tasks, which requires procedural knowledge: “*...if there’s no need for them to have IV fluids running and they’re eating and drinking and then you can make a decision to stop the IV fluids*” (P22 SN:5y).

Finally, participants described autonomy as being demonstrated in exceptional circumstances, such as emergency situations.

### Subtheme: demonstrating autonomy in exceptional circumstances

Nurses’ level of autonomy is situational. Some nursing work is routine, as described above, but the patient’s condition can quickly deteriorate requiring the nurse to take action. Sometimes this involves anticipating the information that the doctor will require, such as an electrocardiogram (ECG). In other cases, it is an independent decision such as giving the patient oxygen. Nurses also need to be more autonomous in the absence of senior professionals, especially on weekends, as gathered from the following comment by a ward manager: “*...my junior sister would make a decision to take out a central line, to take out a catheter and to move a patient onto diet and fluids without referring to a doctor over a weekend*” (P18 WM:18y).

The above opinion was supported by a staff nurse who described autonomy in relation to the staff nurse taking a lead role in decision making and assuming more responsibility on weekends when there was no senior member of staff to offer support. She emphasised that the ward sisters or the sisters in charge ran the ward during the week, but that the staff nurses took a leading role in running the shifts on weekends due to unavailability of more senior nurses to offer support in decision making. One participant stated: “*I would probably take more of an autonomous role of a weekend in a way, of that leadership… the sisters of a week kind of run the shift don’t they, or the nurse in charge*” (P21 SN:2y9m). This comment suggests that autonomy can be turned on and off as necessary rather than being ingrained in practice.

Another two participants corroborated the above comment stating: “*I know there are people who are higher than me, like the site managers or things like that. Sometimes you feel that… when you are in charge of the ward, you make some decisions*” (P17 SN:3y), and “*…so at that time I feel I have made an autonomous decision because I was in charge at the time and I didn’t have somebody else to ask*” (P25 SN:20y).

Additionally, a ward manager described autonomy as making decisions on the spur of the moment when there is lack of constant support. She noted that at times nurses are put in difficult situations to make such decisions, which they would not have made if they had a choice:

*...in this line of work where you don't always have somebody 24 hours a day to back you up… you have to make a decision on the spur of the moment whether you are ready to or not. Sometimes the nurses are put in difficult situations where they don't have a choice, whereas given the choice they probably wouldn’t always make those same decisions.* (P35 WM:15y)

Closely related to nurses having to make decisions on the spur of the moment, participants also identified emergency situations in which autonomous decisions were required to save lives. A participant stated: “*I am able to make on-the-spot decisions about patient care in emergency situations and so on that would need to be made without sort of consultation of a doctor or anything first…*” (P16 SR:4y). Another participant noted that emergency situations sometimes required anticipating and getting the required information as part of acting autonomously: “*…if a patient was poorly ...then I would take it upon myself to take bloods from the patient and cultures and call the doctor ...I wouldn’t have to be told to do that, I would do that myself*” (P32 SR:3y).

Anticipating and providing the relevant information, as part of autonomous practice, was illustrated by another participant in an example of a patient who complained of central chest pain:

*I knew that I needed to act quickly just in case that was an acute cardiology problem… I went ahead and did an ECG because I knew that would be the first thing that they [doctors] would want.* (P44 SR:18y)

Although participants described autonomy in practice as important both on a day-to-day basis and in exceptional circumstances, they recognised that there were boundaries to their practice.

## Theme 5: boundaries around autonomy

Participants discussed boundaries related to autonomy as policies and guidelines that are intended to advise people on how something should be done. The Nursing and Midwifery Council (NMC) Code [23], for example, governs standards of practice for UK nurses and midwives. Participants discussed the impact of these guidelines in terms of working for the benefit of the patient.

### Subtheme: working within boundaries

Nurses’ autonomy operates within strict limits. The most important is the NMC code of practice but there are additional policies, guidelines and protocols specific to the individual Trust. Several respondents mentioned working within their own boundaries and limitations which involve a degree of self-knowledge.

During the interviews, participants disclosed that there were different expectations of nurses at different levels within the profession. They described features of their practice that allowed them, or not, to practice autonomously with specific reference to hierarchy and organisational structure:

*...based on where you are in the nursing... I don’t like to say hierarchy but in the nursing management scheme…my junior nurses I would expect to seek advice from the junior sister that is on. I will make a decision to discharge a patient without recourse to a doctor, whereas my junior nurses might actually say to me ‘Do you think this patient... we can send this patient home?’* (P18 WM:18y)

In addition to hierarchy determining whether or not nurses practice autonomously, the NHS structure was described as influencing the work pressure experienced by nurses on the wards. One staff nurse was of the view that the ability of nurses to practise autonomously was determined by ‘outside forces’ depicting the attitudes of those in NHS managerial positions:

…*it seems to be geared towards the ward. Sometimes I think the pressures come from outside of the ward in regard to this which is kind of outside us. Sometimes it’s not the ward manager or the ward itself, it’s those outside like those in managerial positions that force pressure onto the nurses.* (P6 SN: 3½y)

This perception that nurses’ ability to practice autonomously is determined by the attitudes of those in managerial positions was shared by a ward manager:

*If my manager is very controlling, and I might become very controlling to my staff because I think that’s the way it's supposed to work maybe. Now I have got a little bit more experience but even so it could happen that way because you think well you have to follow the organisation’s way of working. But you might find in another department the manager is not so controlling so you become less controlling isn’t it?* (P13 WM:29y)

Some participants described the importance of working within their own boundaries and limitations which involves self-awareness—the understanding of oneself or one’s motive or character. It also involves nurses understanding their needs, failings, and capabilities in patient care, as can be gathered from the following comments: “*....that you act within your own boundaries*” (P38 WM:16y), “*but also knowing their limitations and when to get help*” (P43 WM:10y), and the “*…need to consider our limitations as well, especially with the patient care*” (P17 SN:3y). Similarly, a ward manager described boundaries in terms of nurses having had nursing training that provided them with the capability to look after patients within that remit:

*That the nurse is being seen to be their own individual professional and have had training as such and therefore they should be able to look after patients within that remit… the training would help you to be an autonomous professional.* (P13 WM:29y)

Further to describing hierarchy and organisational structure and policies as boundaries of autonomous practice, the NMC Code of Conduct was highlighted as a factor that could determine or inhibit autonomy practice: “*...if we fear getting into trouble it’s because it depends on the policy inside the structure of the NHS*” (P1 SN:14y). Others described working within boundaries as: “*Kind of being able to be my own boss following guidelines set down by obviously the Trust and NMC*” (P6 SN:3½y); “*They know that they are needing to work within their Code of Conduct*” (P38 WM:16y); and “*…to be able to practice within the guidelines of the NMC and with your own Trust*” (P9 SN:23y).

### Subtheme: working beyond boundaries

Nurses viewed autonomy as the ability to make decisions and advocate for patients. In some cases, autonomy involved working beyond the boundaries of normal practise or protocols for the patient’s benefit. Some of the participants perceived patient advocacy to exist when nurses were empowered by patients to make decisions on their behalf: “*That you’re autonomous for the patient, that you want to act in their best interest and be their advocate and work in an autonomous way, so without judgement, prejudice…*” (P46 SR:10y); “*making patient focused decisions, so making decisions that are in the best interests of the patient…*” (P19 WM:8y).

Another illustration was provided by a participant who used her initiative, based on knowledge of the patient, to make the decision not to remove the patient’s cannula when the patient had to go to a different hospital for an appointment. Although, she was aware that her decision was against the Trust’s policy, she took responsibility for her action in the best interest of the patient:

*...the policy should be we take the cannula out… I thought I would rather send them with a cannula because he's a very difficult patient to cannulate and he’s on 6 hourly antibiotics, so if he comes back late (within the 6 hours he’ll be back)… they are struggling to put in a cannula and he’ll miss his dose and he really needed his antibiotics because he was a vascular patient.* (P25 SN:20y)

Another participant gave an illustration of acting in the best interest of the patient by cancelling the patient’s transport because safety might be compromised as it was late at night, thus defying the hospital’s policy (i.e. boundaries):

*They say they were going to collect the patient at 8 o’clock…10 o’clock the ambulance said... ‘I don’t think we will be able to get your patient as soon as possible now, maybe if you wait for us, book her in an hour,’ and then I said ‘Well I am not happy at all for my patient to go at that time’ so obviously I had to cancel the discharge.* (P17 SN:3y)

## Theme 6: developing autonomy requires support

This theme is linked to participants’ perception of how autonomy can be developed in the junior members of the nursing staff. During the course of the interviews participants discussed how availability and provision of support in their work environment helped them develop their professional capacity of practising autonomously, as stated by (P1 SN:14y): “*…As long as you have the support, then it’s all right*”. Having support in the work environment was highlighted as an important ingredient for the development and promotion of autonomy. The senior nurses believed that when support in making decisions is provided to the junior nurses, they develop self-confidence and are enabled to practice autonomously. A ward manager illustrated how she supported a staff nurse by reducing the number of patients and pairing her with a more senior nurse:

*I have actually put her into the 8 bedded bay with another staff nurse that’s usually more senior, just so that we can build her confidence… because it's good for her to be able to make decisions on a day-to-day basis…* (P28 WM:17y)

Ward managers described the development of autonomy as receiving support from more senior managers:

*I also feel that my manager encourages me to be autonomous in how I manage the ward, in achieving what needs to be done… So yeah, so I get support from my boss, but I have also not got my boss on top of me all the time, so it's giving me freedom to work within what I know I can do, but also to achieve what I need to achieve.* (P23 WM:28y)

Another participant commented on giving support to nurses who are of a lower grade by

*...trying to encourage and guide my nurses towards autonomous practice, it's almost been an element of stepping back and allowing them to go through their own clinical decision-making process to come from A to B to make a decision for that patient and supporting them to do that.* (P34 WM:11y)

Finally, P28 (WM:17y) sought to “*encourage my nurses to work autonomously on the understanding that I am there to support them if they need to come to me, if they are worried about anything.*”

Participants also described the development of autonomy as a situation whereby a junior nurse receives or seeks confirmation or affirmation that he/she has made an appropriate decision:

*…some decisions you need clarification, just confirmation for, so they would perhaps come to me for ‘Have I done the right thing? I am going to do A, B, C, would you say this is the right thing to do?’ because that’s how they learn… even if they make a mistake... for the ones that made the decision… get them to reflect on whatever that is.* (P23 WM:28y)

One of the participants highlighted the need for acknowledgement as a factor that supports the development of autonomy. This staff nurse emphasised the lack of recognition for nurses’ ability to make autonomous decisions as a hindrance to the development of autonomy, because such a situation may make nurses feel undervalued: “*they [nurses] want to be recognised as being able to make decisions*” (P33 SN:3½y).

Nurses disclosed an unwillingness to take risk or accept responsibility for mistakes due to a fear of criticism or prosecution. Participants stated that staff nurses refrain from making autonomous decisions when they perceive that they might be blamed by their colleagues if they did not make the right decisions:

*They are very good at getting together and talking about A, B or C but they’re not that happy in being that assertive and making a statement or making a point to a senior person... they don't want to put themselves on a pedestal and say ‘Right, I know this because X, Y and Z happened. I know the staff will back me, but they’re not willing to come forward and support me’ - so that’s why nurses don’t like taking big risks because of the implications it may have on their career I suppose.* (P26 SN:26y)

Closely related to the above views, both senior and junior nurses highlighted the difficulty of supporting autonomous nursing practice within a “blame culture”. A ward manager argued that removing the blame culture in the NHS was likely to breed autonomy:

*If you want to breed autonomy with your nurses, you cannot have a blame culture because they are going to make mistakes when they are making their own choices and decisions… there will always be a learning opportunity rather than a blame thing because if you do that they’ll just shut down and won’t make decisions.* (P34 WM:11y)

A more junior staff nurse reinforced this view stating: “*I’d say it’s very good that we get all the responsibility, but I think in some cases that blame is a bit of a problem... so if things go wrong they just blame you even though... while you were making it, your decision was supported, but when something went wrong they say ‘Oh, you did this?’*” (P10 SN:8 m).

In summary, this theme described the various ways in which junior nurses were supported by senior nurses in making autonomous decisions in practice. Participants highlighted the development of confidence in the junior nurses, enabling them to make autonomous decisions when they receive affirmation or confirmation before or after making their decisions. They also highlighted the importance of the absence of a blame culture in order for autonomy to thrive.

## Discussion

Utilising an inductive phenomenological descriptive analysis, six key themes were identified that suggest a lack of consensus or ambivalence amongst the participants about the concept of autonomy and what constitutes autonomous nursing practice. Whilst some nurses understood autonomy as working independently, others viewed it as working in a team. In addition, some participants perceived autonomous practice as carrying out actions based entirely on their own decisions, while others wanted support in the form of clarification or confirmation from more senior staff. Despite these conflicting perceptions, an overarching key finding was that nurses related autonomy to their clinical work and to the immediate work environment of their ward rather than to a wider professional context.

In a UK study, [24] identified a strong association between teamwork and autonomy and revealed that nurses who are more involved in team working exhibited higher levels of autonomy and were more involved in decision making. However, in another British study, [25] identified teamwork as constraints on professional autonomy of nurses. Teamwork was described in the study as both empowering and disempowering. Participants in the study maintained that it could be empowering because several professional groups had to work closely together and make joint decisions, which would make the most powerful individual professionals less powerful, and the less powerful individual more influential; yet, it could be disempowering because the nurses themselves would lose part of their professional autonomy through the inter-professional teamwork.

It was also identified that autonomy expressed through everyday tasks is implied rather than overtly expressed. Although Gagnon and colleagues [3] revealed that autonomy was a topic not openly discussed among nurses. Participants in [26] discussed their ability to organise their work day, set priorities among the tasks, assessments, and personal care, as examples of inherent autonomy in their practice. Likewise, [1] emphasised that performing tasks is an essential part of autonomous nursing practice. However, participants in an American study [27] argued that it is not autonomy when, for example, a nurse decides to advance a patient’s diet from soft to full, or to discontinue IV fluids when a patient is eating and drinking. They maintained that the decision is based on knowledge and assessment, but the nurse is acting on instruction to ‘advance diet as tolerated’; therefore, it is not considered autonomy.

Participants described autonomy as making independent decisions in exceptional situations, such as during emergencies, when junior nurses find themselves to be in charge of the wards on weekends, or when senior members of staff are not available, suggesting that autonomy can be turned off and on rather than an integrated part of nursing. Similarly, [26] revealed that nurses, by default, felt acutely responsible for everything overnight due to the relative absence of other team members, which challenges their scope of practice. These views were supported by [1] where participants identified the theme “to dare” (p. 2231) to express their personal endeavours in challenging situations where there were no standards or routine to follow.

The subtheme *‘Working within boundaries’*, explored the determinants or hindrances to autonomy, such as the hierarchy and organisational structure in the NHS. It has been argued that as long as another unit of the organisation legitimately can veto power, autonomy cannot exist [28]. Kramer and Schmalenberg [29] maintained that an ingredient for autonomous practice at the staff nurse level is a flat, debureaucratised organisational structure. They stated that nurses will not function autonomously, even if they are competent, if they have to ‘go through channels’ to get decisions made. Likewise, [25] described hierarchical decision-making as constraints on nurses’ professional autonomy.

Participants in this study identified several examples where they had breached boundaries for the benefit of the patients. It has been identified [30] that acting as patient advocate may put nurses at personal and professional risk. Building trust in the clinical setting by supporting nursing actions that may be risky, yet are safe, encourages innovative practice and enhances autonomy [31].

It could be argued, based on the findings from this study, that the ability of a nurse to make and act on discretionary decisions is dependent on the level of his/her knowledge, competence, and confidence. It could also be argued that the ability to make discretionary decisions is consistent with the nurse’s scope of practice, as the nurse is equipped with the knowledge required to make such decisions and, therefore, should not need to confirm such decisions with other members of staff. However, the presence of a blame culture is one of the limitations the participants perceived was associated with autonomous decision making. Lewis and Batey [28] stated that decisions and actions in the context of autonomy are the professional’s own; and cannot be shifted to another when the outcomes have been less than favourable.

Removing the NHS blame culture around mistakes is essential to improving patient safety [32]. Keegan [33] equated accountability to responsibility and answerability to authority for one’s actions. Thus, if an individual is prepared to act autonomously, the individual must be prepared to accept that he/she must be answerable for his/her action. Moving from a blame culture to a just culture requires a comprehensive understanding of organisational attributes or antecedents that cause blame or just cultures [34]. Khatri and colleagues [34] maintained that a blame culture is more likely to occur in health care organisations that rely predominantly on hierarchy and compliance-based functional management systems. A just culture is more likely to occur in health organisations that elicit greater employee involvement in decision-making.

### Relevance to clinical practice

There was no mention in these short interviews of acting autonomously within the hospital and being involved in managerial, or higher-level decision-making. However, the research highlighted hierarchy and organisational structure in the NHS as determinants or hindrances to autonomy. It is suggested that the nursing profession in England should adopt a more participative decision-making style, similar to that of America, where nurses emphasise involvement in hospital level committees. Nurses should be more involved in writing protocols and part of hospital boards. The importance of education in supporting and enhancing autonomous practice was highlighted. To breed autonomy, continuous professional developmental courses focussing on clinical skills, autonomy, decision-making, and leadership should be offered to nurses. Some participants implied that autonomy could be turned on and off as necessary, whilst some suggested that autonomous practice is an important element that should be focused on during nursing. Based on these findings, it is suggested that current nursing education should provide opportunities for personal and professional development which would promote autonomous practice in students. We argue that, in the long run, this would ingrain autonomy into practice and improve the professional standing of nursing in England. The nursing profession needs to be more autonomous.

### Limitations

This study was conducted in two NHS hospitals in the South East of England. This makes it difficult to say how typical they are of all acute trusts in England and may limit the generalizability of the findings. The fact that the study’s sample was predominantly women could be a limitation, as findings might have differed if there were more male participants.

## Conclusion

There is a lack of consensus amongst the sample of English practicing nurses regarding the concept of autonomy and what constitutes autonomous nursing practice. There appears to be no set definition of autonomy and interpretations of autonomy were found to be diverse. Importantly, when nurses talked about autonomy, they did not relate it to the achievement of professional status; rather, nurses were clinically focused and limited their discussions of autonomy to the ward team, implying a need for the NHS to adopt a participative decision-making style wherein staff nurses will be more involved in hospital-level decision making.

## Data Availability

The dataset from this study is available from the corresponding author upon reasonable request.

## Abbreviations

16y: 16 years
2y9m: 2 years 9 months
BNF: British National Formulary
CN: Charge Nurse
ECG: Electrocardiogram
EOMII Scale: Essentials of Magnetism II Scale
IV: Intravenous
MDT: Multi-Disciplinary Team
NHS: National Health Service
NMC: Nursing and Midwifery Council
non-US: non-United States
P: Participant
RQ: Research Question
SN: Staff Nurse
SR: Sister
WM: Ward Manager
